# Outcomes of patients with cobalamin C deficiency: A single center experience

**DOI:** 10.1002/jmd2.12179

**Published:** 2020-11-08

**Authors:** Danielle K. Bourque, Lizbeth E. Mellin‐Sanchez, Garrett Bullivant, Vivian Cruz, Anette Feigenbaum, Stacy Hewson, Julian Raiman, Andreas Schulze, Komudi Siriwardena, Saadet Mercimek‐Andrews

**Affiliations:** ^1^ Division of Clinical and Metabolic Genetics, Department of Pediatrics The Hospital for Sick Children Toronto Ontario Canada; ^2^ Department of Pediatrics University of Toronto Toronto Ontario Canada

**Keywords:** cobalamin C, global developmental delay, homocystinuria, methylmalonic acid, newborn screening, stroke

## Abstract

Biallelic variants in *MMACHC* results in the combined methylmalonic aciduria and homocystinuria, called cobalamin (cbl) C (cblC) deficiency. We report 26 patients with cblC deficiency with their phenotypes, genotypes, biochemical parameters, and treatment outcomes, who were diagnosed and treated at our center. We divided all cblC patients into two groups: group 1: SX group: identified after manifestations of symptoms (n = 11) and group 2: NB group: identified during the asymptomatic period via newborn screening (NBS) or positive family history of cblC deficiency (n = 15). All patients in the SX group had global developmental delay and/or cognitive dysfunction at the time of the diagnosis and at the last assessment. Seizure, stroke, retinopathy, anemia, cerebral atrophy, and thin corpus callosum in brain magnetic resonance imaging (MRI) were common in patients in the SX group. Global developmental delay and cognitive dysfunction was present in nine patients in the NB group at the last assessment. Retinopathy, anemia, and cerebral atrophy and thin corpus callosum in brain MRI were less frequent. We report favorable outcomes in patients identified in the neonatal period and treated pre‐symptomatically. Identification of cblC deficiency by NBS is crucial to improve neurodevelopmental outcomes.

**Abbreviations**
AdoCbl: adenosylcobalamin
C3: propionylcarnitine
cbl: cobalamin
cblC: cobalamin C
CC: corpus callosum
FTT: failure to thrive
GDD: global developmental delay
HC: homocystinuria
Hcy: homocysteine
ID: intellectual disability
LD: learning disability
MeCbl: methylcobalamin
Met: methionine
MMA: methylmalonic acid
MRI: magnetic resonance imaging
NB group: patients identified in neonatal period or due to positive family history
NBS: newborn screening
SX group: patients identified after manifestations of symptoms
WM: white matter

## 1 INTRODUCTION

Biallelic variants in *MMACHC* results in the combined methylmalonic aciduria and homocystinuria, called cobalamin (cbl) C (cblC) deficiency. cblC deficiency is the most common disorder of intracellular cobalamin metabolism.^1^, ^2^ Clinical presentation and age of onset of cblC deficiency is variable. Severe forms may present with intrauterine growth restriction, microcephaly, neurologic impairment, cytopenia, hemolytic uremic syndrome, metabolic crises, and dilated cardiomyopathy.^2^, ^3^, ^4^ In contrast, later onset forms may present with thromboembolic events (eg, stroke), psychosis, and progressive encephalopathy.^2^, ^5^ Neurodevelopmental disorders^6^, ^7^, ^8^ and ophthalmologic involvement (ie, maculopathy, retinopathy)^9^, ^10^ are also common.

We performed a retrospective cohort study and included all patients with cblC deficiency diagnosed and followed at our institution. We report 26 patients with respect to their phenotype, genotype, biochemical parameters, and treatment outcomes. Additionally, we compare outcomes for patients identified after manifestation of symptoms (SX group) to those patients identified asymptomatically by either positive newborn screening (NBS) or positive family history of an affected older sibling (NB group) in our study.

## 2 METHODS

This retrospective cohort study was approved by the Research Ethics Board at The Hospital for Sick Children (REB#1000053387). All patients with cblC deficiency were included. Patients were divided into two groups to compare treatment outcomes of patients treated symptomatically and asymptomatically: group 1: SX group: identified after manifestations of symptoms; and group 2: NB group: identified during the asymptomatic period via NBS or positive family history. Patients in both groups were also divided into two groups to assess treatment outcomes based on the age of onset and age at last assessment: (a) SX group: the age at symptom onset <4 years and ≥4 years of age. (b) NB group age at the last assessment <4 years and ≥4 years of age.

Patient charts were reviewed for clinical features, biochemical investigations, molecular genetic investigations, neuroimaging, treatment modalities, and outcomes.

Statistical analysis was conducted using GraphPad Software (San Diego, California) and R software (version 1.2.1335). Results are presented as mean ± SD (range). Unpaired two‐tailed *t*‐tests, Fisher's exact tests, Welch two sample t‐test (parametric test) and Kruskal‐Wallis rank sum test (non‐parametric) were chosen to compare outcomes between groups. A *P*‐value of <.05 was considered to be statistically significant.

## 3 RESULTS

**FIGURE 1:**
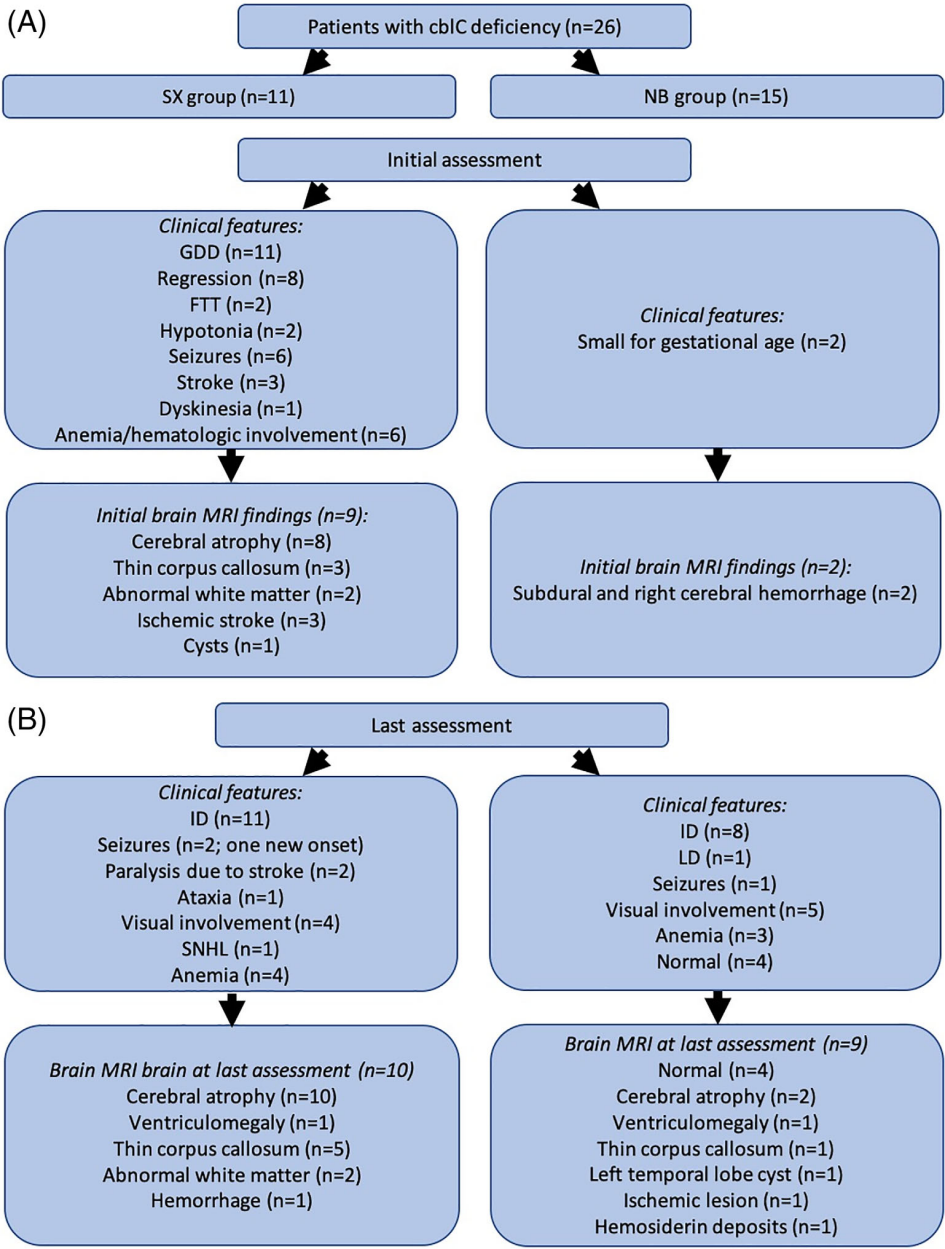
Clinical features of patients with cblC deficiency at the time of diagnosis, A, and at the time of last assessment, B, are depicted. SX group, patients identified after manifestations of symptoms; NB group, patients identified in neonatal period or due to positive family history

There were 26 patients (14 males, 12 females) from 21 unrelated families (five families had two children with cblC) diagnosed with cblC deficiency until April 2016 (time of the Research Ethics Board approval of this study) at our institution. There were 11 patients in the SX group: nine born prior to expanded NBS in 2006 and two were born after NBS, but those two patients had false negative NBS for propionylcarnitine (C3). There were 15 patients in the NB group (13 identified by positive NBS for C3 and two with false negative NBS for C3 with a family history of an affected older sibling). Clinical, biochemical, genotype, and neuroimaging features and treatments and outcomes are summarized in Table 1 (SX group) and Table 2 (NB group). NBS results and hematological results of SX and NB group patients are listed in Table S1. Biochemical investigations are summarized in Table 3. The clinical and radiographic features of patients in the SX and NB groups at the time of diagnosis (Figure 1A) and at the time of last follow up (Figure 1B) are presented in Figure 1.

**TABLE 1:** Clinical features, biochemical features, neuroiamaging and treatments of SX group patients

Patient/sex/ethnicity/age of diagnosis/age last seen	Clinical features initial (onset)/last follow‐up	Initial investigations/Last visit investigations C3/urine MMA/plasma MMA/homocysteine/methionine (umol/L)	*MMACHC* variants	Neuroimaging initial (age)/neuroimaging follow‐up (age)	Treatment^a^
1/F/Italian, Portuguese/1 mo/6.8 y	Neonatal focal seizures, neonatal stroke (1mo)/right HP, Eso, LD, ADHD	C3 = 3.3/0.5 U‐MMA = 644/19 P‐MMA = 4.84/1.6 Hcy = 57/43 Met = 48/25	Cmp HTZ c.271dupA (p.Arg91Lysfs*14)/c.389A > G (p.Tyr130Cys)	Restricted diffisuion in left frontal, insula, temporal, parietal lobes, thalamus (left middle cerebral artery territory ischemic stroke (2w)/Left fronto‐temporo‐parietal encephalomalacia (2 m)	OH‐Cbl = 0.047 Bet = No Car = No FA = 2
2/M/Lebanese/5 mo/13.4 y	GDD,Mic, Hyp, TMA, HUS (2mo)/ID, GTCS, Eso, Nys, right SNHL, RN	C3 = 8.3/1.9 U‐MMA = 272/1645 P‐MMA = 103/22 Hcy = 214/29 Met = 14/28	HMZ c.547_548delGT (p.Val183Thrfs*5)	NP/CA, VM, thin CC (12.1y)	OH‐Cbl = 0.011 Bet = 222 Car = 22 FA = 10
3/M,Chinese/6.3 y/11.7 y	GDD (6‐12mo)/GTCS (6.1 y) stroke (6.1 y), ID	C3 = 4.5/0.5 U‐MMA = 618/3 P‐MMA = 14/2.8 Hcy = 318/40 Met = 5/27	Cmp HTZ c.658_660delAAG (p.Lys220del)/c.609G > A (p.Trp203*)	CA, CeA, increased T2/FLAIR signal in frontal, periventricular, peritrigonal, parietal WM, left ventricul SEC, thin CC (6y)/No change (6.8 y)	OH‐Cbl = 0.13 Bet = 250 Car = No FA = 10
4/F/Indian/8.8 y/12.7 y	Regression, hallucination (8.7 y)/GDD	C3 = 2.7/0.5 U‐MMA = 242/18 P‐MMA = 24/2 Hcy = 241/28 Met = 7/25	HMZ c.394C > T (p.Arg132*)	CA (8.7 y)/NP	OH‐Cbl = 0.116 Bet = 211 Car = 219 FA = 5
5/F/Indian/4.8 y/10.5 y	Regression, focal seizures (3.7 y)/GDD, AD	C3 = 1.1/1.7 U‐MMA = 442/8 P‐MMA = 9.6/2.8 Hcy = 63/27 Met = 24/38	HMZ c.394C > T (p.Arg132*)	CA, thin CC (5 y)/No change (7.2 y)	OH‐Cbl = 0.071 Bet = 131 Car = 18 FA = 10
6/M/Indian/9.3 y/14.9 y	Regression, seizures (7.9 y)/stroke, dysphasia (8.5 y), ID, rigt HP	C3 = 1/1.4 U‐MMA = 150/10 P‐MMA = 15/2.4 Hcy = 52/43 Met = 13/45	HMZ c.394C > T (p.Arg132*)	Left fronto‐parieto‐tempro‐occipital, BG increased T2/FLAIR signal, MCA occlusion ischemic stroke (8.5y)/CA (9 y)	OH‐Cbl = 0.09 Bet = 198 Car = 28 FA = 10
7/F/Pakistani/1.1 y/3.5 y	Regression, FTT, dyskinesia, tremor, Hyp/GDD	C3 = 3.54/2.7 U‐MMA = 854/156 P‐MMA = 40/13 Hcy = 38/36 Met = 9/22	HMZ c.394C > T (p.Arg132*)	CC, delayed myelination, thin CC, subdural effusions (9mo)/CA, thin CC (2 y)	OH‐Cbl = 0.133 Bet = 213 Car = 75 FA = 1
8/M/Italian/6 mo/13.3 y	FTT, encephalopathy (1mo)/GDD, ID, Mic, ASD, ADHD, Eso	C3 = 5.3/0.9 U‐MMA = 1560/32 P‐MMA = 13.6/4.6 Hcy = 190/56 Met = 3/25	Cmp HTZ c.331C > T (p.Arg111*)/c.666C > A (p.Tyr222*)	Internal capsule, frontal PVWM (1mo)/WM atrophy, thin CC (9.4 y)	OH‐Cbl = 0.159 Bet = 215 Car = 29 FA = 10
9/F/European/12.8 y/18 y	Speech delay, cognitive dysfunction (8 y)/regression (12.6 y), RN, motor NeP	C3 = 1.1/1.1 U‐MMA = 20.4/25 P‐MMA = 3.5/6.8 Hcy = 195/88 Met = 0/21	HMZ c.3G > A (p.Met1?)	CA (13.2 y)/mesial temporal increased signal in FLAIR (18 y)	OH‐Cbl = 0.028 Bet = 91 Car = 7.5 FA = 5
10/F/Italian/6.2 y/13.7 y	GDD (1‐2 y)/GTCS (6 y), regression, behavioral problems, ID	C3 = 0.6/0.6 U‐MMA = 103/103 P‐MMA = 5.1/5.1 Hcy = 75/77 Met = 15/31	HMZ c.3G > A (p.Met1?)	NP/NP	OH‐Cbl = 0.0319 Bet = 352 Car = 29 FA = 1
11/F/Indian/10.4 y/18 y	GDD (1‐2 y)/FTT, regression, Mic, ID, tremor, ataxia, GTCS	C3 = 0.9/1.0 U‐MMA = 5558/32 P‐MMA = 28/3.3 Hcy = 184/60 Met = 11/21	HMZ c.394C > T (p.Arg132*)	CA, CeA (10.1 y)/No change (15 y)	OH‐Cbl = 0.0178 Bet = 255 Car = 15 FA = 5

*Note:* Reference ranges for investigations: Propionylcarnitine (C3) (reference <1.1 mmol/L); Methionine (reference 13‐44 mmol/L).

Abbreviations: AD, attention deficit; ADHD, attention deficit hyperactivity; ASD, autism spectrum disorder; Bet, betaine; BG, basal ganglia; CA, cerebral atrophy; Car, carnitine; CC, corpus callosum; CeA, cerebellar atrophy; Cmp HTZ, compound heterozygous; Eso, esotropia; FA, folinic acid; FTT, failure to thrive; GDD, global developmental delay; GTCS, generalized tonic‐clonic seizures; Hcy, homocysteine; HMZ, homozygous; HP, hemiparesis; HUS, hemolytic uremic syndrome; Hyp, hypotonia; LD, learning disability; Met, methionine; Mic, microcephaly; NeP, neuropathy; NP, not performed; Nys, nystagmus; OH‐Cbl, hydroxycobalamine; P‐MMA, plasma methylmalonic acid; PVWM, periventricular white matter; RN, retinopathy; SNHL, sensorineural hearing loss; SEC, subependymal cyst; TMA, thrombotic microangiopathy; U‐MMA, urine methylmalonic acid; VM, ventriculomegaly; WM, white matter.

^a^ Hydroxycobalamine, betaine and carnitine doses were provided as mg/kg/d dose. Folinic acid was provided as mg/d dose.

**TABLE 2:** Clinical features, biochemical features, neuroiamaging and treatments of NB group patients

Patient/sex/ethnicity/age last seen	Clinical features initial at birth /last follow‐up	Initial investigations/last visit investigations C3/urine MMA/plasma MMA/homocysteine/methionine (umol/L)	*MMACHC* variants	Neuroimaging initial (age)/neuroimaging follow‐up (age)	Treatment^a^
1/F/Afghani/5 y	IUGR, Mic/GDD, Eso, MP	C3 = 22/8.4 U‐MMA = 231/78 P‐MMA = 33/9.6 Hcy = 111/37 Met = 11/33	Cmp HTZ c.271dupA (p.Arg91Lysfs*14)/c.388_390delTAC (p.Tyr130del)	NP/CA, CeA (2.7 y)	OH‐Cbl = 0.29 Bet = 255 Car = 83 FA = 2
2/M/European‐Indian/4.4 y	Asym/GDD	C3 = 9.9/1 U‐MMA = 1623/27 P‐MMA = 75/1.9 Hcy = 179/15 Met = 5/19	Cmp HTZ c.271dupA (p.Arg91Lysfs*14)/c.394C > T (p.Arg132*)	NP/NP	OH‐Cbl = 0.29 Bet = 251 Car = 151 FA = 5
3/F/Chinese/10 mo	Asym/FTT	C3 = 16/1.5 U‐MMA = 541/45 P‐MMA = 58/2.5 Hcy = 220/24 Met = 7/26	Cmp HTZ c.609G > A (p.Trp203*)/c.658_660delAGG (p.Lys220del)	Increased signal in left thalamus, bilateral subcortical WM, right cerebellar hemorrhage, right parietal subdural hematoma (2w)/Left thalamic ischemic lesion (10mo)	OH‐Cbl = 0.1 Bet = 206 Car = 45 FA = 5
4/M/Chinese/4.8 y	Asym/N	C3 = 11/1.5 U‐MMA = 877/91 P‐MMA = 38/9.4 Hcy = 363/50 Met = 11/16	HMZ c.609G > A (p.Trp203*)	NP/NP	OH‐Cbl = 0.055 Bet = 217 Car = No FA = 5
5/M/Indian/4.4 y^b^	Asym/N	C3 = 9.3/1.3 U‐MMA = 156/24 P‐MMA = 17/4.2 Hcy = 119/28 Met = 17/29	HMZ c.394C > T (p.Arg132*)	NP/N (1.2 y)	OH‐Cbl = 0.117 Bet = 223 Car = 17 FA = 2
6/M/European/3.9 y	Asym/GDD, Eso	C3 = 22/3.5 U‐MMA = 209/57 P‐MMA = 105/6.6 Hcy = 287/32 Met = 4/25	HMZ c.271dupA (p.Arg91Lysfs*14)	NP/left temporal lobe cycst (3 y)	OH‐Cbl = 0.3125^c^ Bet = 262 Car = 50 FA = 5
7/M/Italian/3.6 y	Asym/speech‐language delay	C3 = 12/3 U‐MMA = 20 270/205 P‐MMA = 87/7.6 Hcy = 197/26 Met = 16/27	HMZ c.271dupA (p.Arg91Lysfs*14)	NP/NP	OH‐Cbl = 0.33 Bet = 250 Car = 50 FA = 2
8/M/Afghani/7.6 y	Asym/GDD	C3 = 13/1.7 U‐MMA = 1350/10 P‐MMA = 110/2.7 Hcy = 299/39 Met = 6/23	HMZ c.388_390delTAC (p.Tyr130del)	NP/N (5.4 y)	OH‐Cbl = 0.31 Bet = 237 Car = 23 FA = 10
9/M/Afghani/5.5 y	Asym/GDD	C3 = 7.8/2.9 U‐MMA = 397/23 P‐MMA = 182/4.5 Hcy = 195/37 Met = 15/21	HMZ c.388_390delTAC (p.Tyr130del)	NP/CA (5 y)	OH‐Cbl = 0.375 Bet = 256 Car = 26 FA = 5
10/F/Pakistani/3.7 y	Asym/GDD, Nys, MP, RN	C3 = 10/6.1 U‐MMA = 1479/443 P‐MMA = 190/16 Hcy = 293/34 Met = 9/35	HMZ c.551_554dupCTAC (p.Arg186Tyrfs*4)	NP/N (1.7 y)	OH‐Cbl = 0.16 Bet = 246 Car = 66 FA = 5
11/F/Pakistani/1.5 y	Asymp/N	C3 = 3/1.7 U‐MMA = 216/33 P‐MMA = 29/3.9 Hcy = 122/33 Met = 8/20	Cmp HTZ c.271dupA (p.Arg91Lysfs*14)/c.394C > T (p.Arg132*)	NP/NP	OH‐Cbl = 0.091 Bet = 261 Car = 44 FA = 5
12/M/Pakistani/2.4 y	SGA/speech delay	C3 = 4.6/1.6 U‐MMA = 357/32 P‐MMA = 33/3.8 Hcy = 217/33 Met = 5/22	Cmp HTZ c.271dupA (p.Arg91Lysfs*14)/c.394C > T (p.Arg132*)	Increased signal in fronto‐temporal WM in T2 (2wks)/NP	OH‐Cbl = 0.071 Bet = 259 Car = 48 FA = 5
13/M/European‐Guyanese/5.6 y	Asmp/GDD, seziures, Eso	C3 = 7.6/2.4 U‐MMA = 2165/67 P‐MMA = 56/5.6 Hcy = 192/22 Met = NP/9	Cmp HTZ c.271dupA (p.Arg91Lysfs*14)/c.609G > A (p.Trp203*)	Rigth ventricule septation, hemosiderin (1 m)/right VM, thin CC (4 y)	OH‐Cbl = 0.05 Bet = 252 Car = 47 FA = 5
14/F/Pakistani/9 mo^b^	Asymp/N	C3 = 1.9/1.7 U‐MMA = 71/129 P‐MMA = 12.5/6.4 Hcy = 39.5/47 Met = 29/29	HMZ c.394C > T (p.Arg132*)	NP/NP	OH‐Cbl = 0.117 Bet = 209 Car = 48 FA = 1
15/M/Chinese/7.4 y	Asym/ASD	C3 = 12/2.9 U‐MMA = 810/118 P‐MMA = 167/18 Hcy = 433/53 Met = 9/37	Cmp HTZ c.609G > A (p.Trp203*)/c.658_660delAAG (p.Lys220del)	NP/N (1.2 y)	OH‐Cbl = 0.104 Bet = 249 Car = 50 FA = 2

Abbreviations: AD, attention deficit; ADHD, attention deficit hyperactivity; ASD, autism spectrum disorder; Asym, asymptomatic; Bet, betaine; BG, basal ganglia; CA, cerebral atrophy; Car, carnitine; CC, corpus callosum; CeA, cerebellar atrophy; Cmp HTZ = compound heterozygous; Eso, esotropia; FA, folinic acid; FTT, failure to thrive; GDD, global developmental delay; GTCS, generalized tonic‐clonic seizures; Hcy, homocysteine; HMZ, homozygous; HP, hemiparesis; HUS, hemolytic uremic syndrome; Hyp, hypotonia; IUGR, intrauterine growth retardation; LD, learning disability; MP, maculopathy; Met, methionine; Mic, microcephaly; N, normal; NeP, neuropathy; NP, not performed; Nys, nystagmus; OH‐Cbl, hydroxycobalamine; P‐MMA, plasma methylmalonic acid; PVWM, periventricular white matter; RN, retinopathy; SGA, small for gestational age; SNHL, sensorineural hearing loss; SEC, subependymal cyst; TMA, thrombotic microangiopathy; U‐MMA, urine methylmalonic acid; VM, ventriculomegaly; WM, white matter.

^a^ Hydroxycobalamine, betaine and carnitine doses were provided as mg/kg/d dose. Folinic acid was provided as mg/d dose.

^b^ Positive family history in older sibling.

^c^ 4 d/wk.

**TABLE 3:** Biochemical investigations at the time of NBS retrieval and diagnosis and at last assessment for patients with cblC deficiency

	NBS							
	C3 μmol/L, mean ± SD (range)	C3/C2, mean ± SD (range)	Age at collection (mo/y), mean ± SD (range)	Plasma C3, μmol/L, mean ± SD (range)	Hcy, μmol/L, mean ± SD (range)	Met, μmol/L, mean ± SD (range)	Plasma MMA, μmol/L, mean ± SD (range)	Urine MMA, μmol/mol creatinine mean ± SD (range)
SX group cblC (n = 11) at diagnosis	N/A	N/A	65 ± 54 m (1‐152)	2.9 ± 2.4 (0.6‐8)	148 ± 94 (38‐318)	14 ± 13 (0‐48)	24 ± 28 (5‐103)	951 ± 1589 (20‐5558)
SX group cblC (n = 11) at last follow up			12.4 ± 4.3 y (3.5‐18)	1.2 ± 0.7 (0.5‐2.7)	48 ± 20 (27‐88)	28 ± 7 (21‐45)	6.1 ± 6.2 (1.6‐22)	186 ± 486 (3‐1645)
SX group at diagnosis vs last follow up				*P* = .04	*P* = .006	*P* = .006	*P* = .03	*P* = .17
NB group cblC (n = 15) at diagnosis	10 ± 5 (3‐19)	0.4 ± 0.2 (0.1‐0.9)	0	11 ± 5.9 (1.9‐22)	218 ± 103 (39‐433)	11 ± 6 (4‐29)	79 ± 59 (12‐190)	2050 ± 5080 (71‐20 270)
NB group cblC (n = 15) at last follow up			4.1 ± 2.1 y (0.75‐7.6)	2.8 ± 2.0 (1‐8.5)	34 ± 10 (15‐53)	27 ± 6.7 (16‐37)	6.8 ± 4.7 (1.9‐18)	92 ± 110 (10‐205)
NB group at diagnosis vs last follow up				*P* < .0001	*P* < .0001	*P* < .0001	*P* = .0002	*P* = .15
SX group vs NB group at diagnosis				*P* < .001	*P* = .09	*P* = .48	*P* < .001	*P* = .49
SX group vs NB group at last follow up				*P* = .02	*P* = .027	*P* = .72	*P* = .75	*P* = .47

*Note:* Reference ranges: NBS C3 0.68 to 3.9, C3/C2 < 0.23; plasma C3 < 1.08; hcy: 5 to 15; methionine 13 to 44; plasma MMA <0.37; urine MMA <5.

Abbreviations: C3, propionylcarnitine; cblC, cobalamin C; hcy, homocysteine; met, methionine; MMA, methylmalonic acid; NB group, patients identified in neonatal period or due to positive family history; NBS, newborn screening; SX group, patients identified after manifestations of symptom.

All patients had molecular genetic confirmation of cblC deficiency. Sequencing of *MMACHC* identified 11 pathogenic or likely pathogenic variants (10 known and one novel; nine truncating and two missense). Ten patients had homozygous and 16 patients had compound heterozygous variants. The most common variant was the pathogenic c.394C > T (p.Arg132*) variant, which was identified in the homozygous state in 5 of 11 patients in the SX group and in 2 of 15 patients in the NB group, and in the heterozygous state in 3 of 15 patients in the NB group. All of those patients had Indian or Pakistani ethnic background. The second most common variant was also pathogenic, c.271dupA (p.Arg91Lysfs*14) variant, which was identified in the heterozygous state in 1 of 11 patient in the SX group, in the homozygous state in 2 of 15 patients in the NB group, and in the heterozygous state in 2 of 15 patients in the NB group. Parents were heterozygous for the variants identified in their children. The ethnic background of those patients was panethnic including Afghani, Pakistani, Northern, and Southern European. All variants are listed in Tables 1 and 2.

All patients were treated with parenteral hydroxycobalamin (average dose of 3 mg/d, range 1‐7.5 mg/d) and folic acid (average dose of 5 mg/d, range 1‐10 mg/d) (Table S1). All patients (except one symptomatic patient) were treated with betaine. In the SX group, the average betaine dose was 194 mg/kg/d (range 90‐352 mg/kg/d) and in the NB group the average betaine dose was 242 mg/kg/d (range 209‐262 mg/kg/d). l‐Carnitine was supplemented in nine out of 11 of the SX group patients and in 14 out of 15 of the NB group patients at an average dose of 50 mg/kg/d (range 7.5‐219 mg/kg/d). None of the patients had a protein‐restricted diet. There were no significant treatment differences between the SX and NB groups.

### 3.1 Clinical features and treatment outcomes of cblC patients in the SX group (n = 11)

The clinical and radiographic features at the time of diagnosis (Figure 1A) and at the time of last follow up (Figure 1B) are presented in Figure 1. The average age of symptom onset was 37.3 ± 44 months SD (range 1‐104 months). The diagnosis was confirmed at an average age of 65 ± 54 months SD (range 1‐154 months). The age of stroke was neonatal, 6 and 8 years in three patients, respectively. Hematologic abnormalities included megaloblastic anemia (n = 5) and thrombocytopenia (n = 1). Renal involvement was demonstrated by hemolytic uremic syndrome (n = 1) and thrombotic microangiopathy (n = 1) at the time of diagnosis.

At the last assessment, the average age of patients in the SX group was 12.5 ± 4.3 years SD (range 3.5‐18 years). Two patients were transferred to an adult metabolic center at 18 years of age. Five patients (out of six) became seizure free, and one patient developed a new onset seizure. Hypotonia improved in two patients. One patient developed thrombotic microangiopathy confirmed via kidney biopsy.

Eight patients in the SX group had onset of symptoms <4 years of age (five of those <1 year of age). The average time between the symptom onset and the diagnosis was 2.6 years in this subgroup. Three patients had a homozygous c.394C > T variant (onset 9 months, 1‐2 years, and 3.7 years, respectively).

Three patients in the SX group had onset of symptoms >4 years of age. The average age of onset was 8.2 years and the average time between the symptom onset and the diagnosis was 2.1 years. Two of these patients had homozygous c.394C > T variants.

Ten out of 11 patients in the SX group were older than 4 years of age at the time of last assessment.

### 3.2 Clinical features and treatment outcomes of cblC patients in the NB group (n = 15)

The clinical and radiographic features at the time of diagnosis (Figure 1A) and at the time of last follow up (Figure 1B) are presented in Figure 1. At the time of last follow up, the average age was 4.1 ± 2.1 years SD (range 9 months −7.6 years).

Seven patients were <4 years at the last assessment. Normal outcome was present in two patients and maculopathy in one patient. Global developmental delay (GDD) and cognitive dysfunction (n = 2) or speech delay (n = 2) were present in four patients. Anemia was present in one patient. Failure to thrive was present in one patient. None of the patients had seizures.

Eight patients were >4 years of age at the last assessment. Normal outcome was present in two patients and maculopathy in one patient. GDD and cognitive dysfunction was present in six patients. Seizure was present in one patient.

### 3.3 Comparison of biochemical features between the SX and NB groups at diagnosis and on treatment at the last assessment

We summarized biochemical investigations and compared SX and NB groups at the time of initial diagnosis prior to initiation of the treatment in Table 3.

At the time of the diagnosis, 5 out of 11 patients in the SX group had normal plasma propionylcarnitine (C3), whereas all patients in the NB group (n = 15) had an elevated plasma C3 (range 1.9‐22 μmol/L). Six out of 11 patients in the SX group had normal methionine, whereas 11 of the NB group patients had low methionine levels (Table 3). The plasma C3 and methylmalonic acid (MMA) levels were significantly higher in the NB group than the SX group. Other biochemical studies were not significantly different between the two groups.

All cblC deficiency patients had statistically significant improvement in their biochemical markers, with the exception of urine MMA levels on treatment (Table 3). The plasma C3 levels were significantly higher in the NB group compared to SX group on treatment, despite the fact that there was no significant difference between the total and free carnitine levels between the two groups. Homocysteine levels were significantly higher in the SX group.

### 3.4 Comparison of genotype and clinical and biochemical treatment outcomes

None of the patients with the c.394C > T (p.Arg132*) variant had developed maculopathy or retinopathy at the time of their last assessment.

In patients with a homozygous or compound heterozygous c.394C > T (p.Arg132*) variant (n = 10), the average homocysteine level was 12.49 ± 15 SD (range 15‐60), whereas in patients with other genotypes (n = 16), the average homocysteine level was 42.94 ± 18.4 SD (range 22‐88) at the last assessment. There was no statistically significant difference (Welch two sample *t*‐test [parametric test] *P*‐value = .2032 and Kruskal‐Wallis rank sum test [non‐parametric] *P*‐value = .4114) between patients with a homozygous or compound heterozygous c.394C > T (p.Arg132*) variant, and patients with other genotypes in homocysteine levels on treatment.

In patients with a homozygous or compound heterozygous c.394C > T (p.Arg132*) variant, average plasma MMA level was 4.49 ± 3.48 SD (range 1.9‐13), whereas in patients with other genotypes, the average plasma MMA level was 7.57 ± 5.87SD (range 1.6‐22) at the last assessment. There was no statistically significant difference (Welch two sample *t*‐test [parametric test] *P*‐value = .1067 and Kruskal‐Wallis rank sum test [non‐parametric] *P*‐value = .1534) between patients with a homozygous or compound heterozygous c.394C > T (p.Arg132*) variant, and patients with other genotypes in homocysteine levels on treatment.

We also looked at the genotype, homocysteine level and hydroxycobalamin dose. In patients with a homozygous c.394C > T (p.Arg132*) variant, the hydroxycobalamin dose range was 0.0178 to 0.133 mg/kg/d and the homocysteine level range was 27 to 60. Interestingly, the highest homocysteine was found in the patient with the lowest hydroxycobalamin dose. Two patients with a homozygous c.271dupA (p.Arg91Lysfs*14) variant had a similar dose and homocysteine levels. In all other patients with different genotypes, the hydroxycobalamin dose range was 0.011 to 0.375 mg/kg/d and the homocysteine level range was 15 to 88. The lowest homocysteine (15) was reported in the patient with 0.29 mg/kg/d hydroxycobalamin dose. Due to the different doses and genotypes, we were not able to apply any statistical methods.

### 3.5 Comparison of outcomes in the SX and NB groups

Interestingly, GDD, cognitive dysfunction, seizure history, stroke history seemed to be more common in the SX group then the NB group. Also cerebral atrophy and thin corpus callosum in brain MRI seemed to be more common in the SX group then the NB group. Outcomes seemed better in the NB group of cblC deficiency patients, compared to the SX group of cblC deficiency patients, both at the time of diagnosis and at the time of last assessment.

## 4 DISCUSSION

We present outcomes of 26 patients with disorders of intracellular cobalamin metabolism as a retrospective cohort study including small number of patients. Early treatment seems to improve clinical outcomes. In the NB group, there seems to be no significant disease progression during follow up. Initiation of the treatment in the neonatal period seems to result in better neurodevelopmental outcomes and less cerebral atrophy in brain MRI in the NB group compared to the SX group. Anemia and retinopathy were not preventable with the early treatment in SX and NB groups. When comparing siblings, identified by symptoms and positive family history, the latter had normal outcomes with early treatment (ages 9 months and 4.5 years). We did not find statistically significant differences between genotype and homocysteine and MMA levels as a response to treatment. Our limited data support a beneficial effect of early treatment to achieve favorable outcomes.

The two most common pathogenic variants were c.394C > T (p.Arg132*) and c.271dupA (p.Arg91Lysfs*14) in *MMACHC* (see Table S1). The pathogenic c.394C > T (p.Arg132*) variant is reported frequently in Indian and Middle Eastern populations and is associated with a later disease onset.^11^, ^12^ Approximately 25% of patients with this variant present between 4 and 10 years old and about 50% of patients present between 10 and 20 years old.^11^, ^12^ Interestingly, three of the SX patients with homozygous c.394C > T (p.Arg132*) variant had early age of onset in our study cohort in contrast to previous reports. NBS was reported to be negative in patients with this variant previously.^13^, ^14^ The two NB patients (phenotypically normal at their last follow up at 9 months and 4.4 years of age) with a homozygous c.394C > T variant were identified via positive family history but had a negative NBS. In our study, the phenotype associated with a homozygous c.394C > T ranged from asymptomatic to symptomatic. The pathogenic c.271dupA (p.Arg91Lysfs*14) variant is the most frequent variant in Caucasians, and is associated with an early disease onset (<1 year).^12^ Outcomes of Chinese patients with homozygous c.609G > A *MMACHC* variant reported recently in several studies.^15^, ^16^, ^17^ In the largest study, neurodevelopmental outcomes were normal in 10 patients identified by NBS and treated from the age of 15 days.^15^ The c.609G > A *MMACHC* variant was identified in five patients (either homozygous or compound heterozygous, one patient from the SX group and four patients from the NB group) in our study. Only five patients in the NB group had normal neurodevelopmental outcome in our study. Although our patient numbers are small, there seems to be no apparent phenotype and genotype correlation in our study compared to the patients reported in the literature with different *MMACHC* variants.

Several recent studies reported neurodevelopmental outcomes in children identified with cblC deficiency on NBS.^6^, ^7^, ^8^, ^18^ Weisfeld‐Adams described 13 patients with early onset disease (oldest 7 years of age), who had GDD/ID (n = 11), hypotonia (n = 11), nystagmus (n = 8), and thin corpus callosum and white matter abnormalities in brain MRI (n = 9)^6^ Hypotonia and nystagmus were more common in that study compared to our study cohort. Interestingly, 11 of those patients had a protein restricted diet; whereas none of our patients had a protein restricted diet. It is unclear if a protein restricted diet alters the disease progression in cblC deficiency. Ahrens‐Nicklas described 12 patients who had feeding difficulties (83%) and anemia (17%) at the time of NBS retrieval.^7^ At the time of their last visit (mean age 6.6 ± 5.4 years SD, range 7 months to 16 years), 87% of those patients had GDD/ID and 75% had retinopathy. Peak homocysteine level was in the neonatal period in the majority of those patients, and study investigators reported this as the strongest predictor of neurodevelopmental outcomes. Homocysteine levels were highest at the time of the diagnosis in our study cohort. Patients with peak homocysteine levels <200 μmol/L seem to have more severe phenotype compared to patients with homocysteine levels 300 to >400 μmol/L based on our observation. In a recent study, 18 patients with cblC deficiency underwent neurodevelopmental assessments. The study showed progressive deterioration of the neurodevelopment, identified after 24 months of age.^8^ It seems that our patient outcomes may be more favorable compared to previous studies.

Interestingly, we had two patients with cblC deficiency in the SX group who had normal NBS for C3 and identified symptomatically by metabolic investigations. Their siblings were diagnosed in the neonatal period due to their positive family history of cblC deficiency. Their NBS for C3 was also negative. Thirteen patients were identified by positive NBS for C3 in the NB group. Despite cblC deficiency is not a primary target NBS disease in our Provincial Newborn Screening Program, positive NBS for C3 allowed us to diagnose and treat patients with cblC deficiency in the neonatal period to improve neurodevelopmental outcomes. It seems that C3 is not able to identify all patients with cblC deficiency via NBS. In addition to C3, homocysteine should be added into NBS, to identify all patients with cblC deficiency in the neonatal period.

Our study has several limitations: (a) This is a retrospective chart review study and patients were followed over a period of 18 years by different metabolic physicians who may have slightly different practice styles; (b) There was no prospective and objective neuropsychological evaluation to assess neurodevelopmental and neurocognitive outcomes and to compare treatment outcomes between the SX group and NB group of cblC patients; (c) Some of the patients in the NB group had no clinical features during the last assessment; however, their average age was 2.8 years (range 9 months to 4.8 years). It may be still too early to see neurodevelopmental delays in those patients; (d) There were several additional patients diagnosed after our study period ended in April 2016; however, they were not included into the study as our Research Ethics Board only approved this retrospective study up to April 2016. (e) Our data include different treatment regimen, different doses of hydroxycobalamin and different times for the initiation of treatment and does not allow us to compare the groups, genotypes and different doses of hydroxycobalamin. Despite these limitations, the number of patients from a single metabolic center and their outcomes are valuable and add additional knowledge regarding cblC deficiency.

In summary, we present 26 patients with cblC deficiency and their treatment outcomes in details in our study. Due to small number of patients with different genotypes and different hydroxycobalamine dose, it is difficult to compare outcomes of patients identified symptomatically or by NBS. There might be favorable outcomes in patients identified and treated in the neonatal period, however large number of patients with application of objective outcome measures would be helpful to show, if identification of patients by NBS would improve neurodevelopmental outcomes.

## CONFLICT OF INTEREST

Danielle K. Bourque, Lizbeth E. Mellin‐Sanchez, Garrett Bullivant, Vivian Cruz, Anette Feigenbaum, Stacy Hewson, Julian Raiman, Andreas Schulze, Komudi Siriwardena, Saadet Mercimek‐Andrews declare that they have no conflict of interest.

## AUTHOR CONTRIBUTIONS

Saadet Mercimek‐Andrews pertinent aspects of the planning, conduct, and reporting of the work described in the article. Danielle K. Bourque, Lizbeth E. Mellin‐Sanchez, Garrett Bullivant, Vivian Cruz, Anette Feigenbaum, Stacy Hewson, Julian Raiman, Andreas Schulze and Komudi Siriwardena conduct, and reporting of the work described in the article. All authors critically reviewed approved the final version of the manuscript.

## INFORMED CONSENT

All procedures followed were in accordance with the ethical standards of the responsible committee on human experimentation (institutional and national) and with the Helsinki Declaration of 1975, as revised in 2000 (5). Institutoional Research Ethics Board at The Hospital for Sick Children approved the study (REB#1000053387) as a retrospective study. The study is approved without individual informed consents as per our Institutional Research Ethics Board requirements.

## ANIMAL RIGTHS

This article does not contain any studies with animal subjects performed by the any of the authors.

## Supporting information


**Table S1.** NBS and hematogical results of the SX and NB group of patients with cblC deficiency.
